# Influence of Laser Processing Parameters on Surface Roughness and Color Formation in the Marked Zone

**DOI:** 10.3390/ma18215037

**Published:** 2025-11-05

**Authors:** Lyubomir Lazov, Nikolay Angelov, Emil Yankov, Tsanko Karadzhov, Dimcho Pulov, Dimitar Dichev

**Affiliations:** 1 Engineering Center, RTU Rezekne Academy, Atbrivosanas Aleja 115, 4601 Rezekne, Latvia; emil.yankov@rtu.lv; 2 Department of Mathematics, Informatics and Natural Sciences, Technical University of Gabrovo, 5300 Gabrovo, Bulgaria; angelov_np@abv.bg; 3 Department of Machine and Precision Engineering, Technical University of Gabrovo, 4 H. Dimitar Str., 5300 Gabrovo, Bulgaria; karadjov_st@abv.bg (T.K.); dpulov@abv.bg (D.P.); dichevd@tugab.bg (D.D.); 4 Center of Competence “Smart Mechatronic, Eco- and Energy-Saving Systems and Technologies”, Technical University of Gabrovo, 5300 Gabrovo, Bulgaria

**Keywords:** laser color marking, AISI 304, raster step, speed, frequency, roughness

## Abstract

This study investigates the influence of laser processing parameters on the surface roughness and color formation of AISI 304 stainless steel. Experiments were conducted to explore how raster step, scanning speed, frequency, linear energy density, and overlap coefficient affect the surface characteristics of laser-marked zones. It was found that increasing the raster step from 20 µm to 80 µm led to a consistent increase in surface roughness (from 1.23 µm to 1.47 µm at 20 kHz and 25 mm/s), accompanied by a shift in color from dark brown to lighter yellow hues. In contrast, increasing scanning speed (from 25 mm/s to 125 mm/s) caused a nonlinear reduction in roughness (e.g., from 1.23 µm to 0.76 µm at 20 kHz and Δ*x* = 20 µm), resulting in a lighter surface color. Frequency was identified as a critical factor; increasing it from 20 kHz to 100 kHz resulted in a threefold decrease in roughness (from 1.23 µm to 0.25 µm at 20 µm raster step and 125 mm/s), which correlated with a shift to brighter yellow tones. Higher linear energy density values (1.60–8.00 J/cm) increased roughness and darkened the surface color, while higher overlap coefficients produced the opposite trend. The study highlights the relationship between surface nanostructuring and the formation of stable interference colors, providing quantitative parameters for achieving desired chromatic effects. These findings establish a basis for the industrial application of laser color marking, where both aesthetic differentiation and functional enhancements—such as corrosion resistance, hydrophobicity, and antibacterial properties—are essential. Future research will focus on quantitatively evaluating the functional properties, including corrosion resistance, hydrophobicity, and durability, of the colored surfaces produced under optimized parameters. This research aims to further develop laser marking as a foundational tool for both aesthetic and functional surface engineering.

## 1. Introduction

Laser processing techniques, such as laser color marking (LCM), have gained significant attention in recent years due to their ability to produce aesthetic, functional, and durable markings on metal surfaces. One of the most frequently studied materials for laser surface modification is AISI 304 stainless steel, a widely used alloy in various industries due to its excellent corrosion resistance, mechanical properties, and versatility. This material is especially prevalent in the food, chemical, medical, and aerospace industries, where stainless steel components are often exposed to extreme conditions, including high temperatures, corrosive environments, and mechanical wear [1]. The material’s aesthetic appeal, due to its ability to maintain its luster and resistance to tarnishing, makes it ideal for high-end appliances and visible applications, such as in architectural surfaces, medical devices, and decorative elements [2]. The ability to achieve precise and reproducible markings, such as color variations and identification codes, is crucial for traceability, branding, and functional applications in these sectors [3].

In particular, AISI 304 stainless steel finds broad use in automotive parts, kitchen equipment, pharmaceutical containers, and aircraft components, where the combination of strength and resistance to oxidation is critical [4]. However, despite its widespread application, the laser surface treatment of AISI 304 presents significant challenges. The process of laser color marking on stainless steel is primarily driven by the formation of oxide layers on the surface. These layers produce different colors due to light interference upon reflection, with the specific color determined by variations in oxide layer thickness. Factors such as laser power, scanning speed, pulse frequency, and hatch spacing directly influence the oxide layer formation, surface roughness, and color outcomes [5,6,7]. These complex relationships, while pivotal to achieving consistent and high-quality marks, are not fully understood, particularly in terms of how the interaction of these parameters affects the surface morphology and color consistency [8].

Nanosecond pulsed lasers have emerged as a key tool for surface treatment due to their ability to induce precise thermal and optical effects on materials, including oxide layer formation and color generation. These lasers, with pulse durations in the range of nanoseconds, provide a highly controlled thermal input that is suitable for generating thin oxide films without significantly melting or deforming the underlying material. Studies have demonstrated that nanosecond lasers are particularly effective for color marking on metals like stainless steel, as they allow for fine control of pulse energy and fluence, which directly impacts the thickness and uniformity of the oxide layer, and thus the resulting color formation [9,10,11]. In addition, laser parameters, including pulse frequency and scanning speed, are critical for achieving stable and reproducible color marks, which remain a challenge in industrial applications [12,13].

A major concern in the industrial application of laser color marking is the reproducibility of the marking process, especially under the high-throughput conditions required in manufacturing environments. To achieve uniform, durable, and aesthetic color in industrial settings, a deep understanding of how processing parameters affect the roughness and optical properties of laser-marked surfaces is essential. Moreover, the energy input, thermal effects, and material properties must be precisely controlled to ensure the formation of high-quality oxide layers without undesirable side effects, such as excessive roughness or inconsistent color formation [14,15,16,17,18].

The purpose of this study is to analyze the influence of some key technological parameters—processing speed *v*, raster step Δ*x*, frequency *ν*, linear energy density *E_l_*, scan overlap coefficient *k_soc_*—on the roughness *R_a_* and color formation during laser color marking of AISI 304 stainless steel samples using a nanosecond laser. Since the color effect of the processed area is primarily due to the diffraction of light from periodic surface structures and oxide layers, the laser raster marking process is examined with particular attention to how these parameters influence the surface morphology and oxide layer growth. These processing parameters are interrelated and also directly affect the energy factors of the process, such as the linear pulse density and the energy density in the treated zone. Understanding these relationships provides essential insights into the initiation and control of laser-induced coloration, with the aim of achieving stable, reproducible, and industrially viable laser marking outcomes.

## 2. Material, Equipment, and Methods

### 2.1. Material: AISI 304 Stainless Steel

The selected material for the experiments was AISI 304 stainless steel, one of the most widely used and versatile grades of stainless steels. It combines excellent corrosion resistance, weldability, and mechanical properties with relatively low cost, making it suitable for applications in the food, chemical, medical, aerospace, and household industries. Typical applications include tanks for liquids and chemicals, kitchen and pharmaceutical equipment, and components in machinery and microelectronics.

The chemical composition and physical properties of the steel are presented in Table 1 and Table 2, respectively.

### 2.2. Laser System

During the study of the marking process, the laser intensity is controlled and scanned based on pre-selected marking parameters, which are edited using a graphical tool. The experiments were conducted using a pulsed optical laser system, specifically designed for high-precision marking applications. The laser operates in the near-infrared range (*λ* = 1064 nm) and offers excellent beam quality (*M*^2^ < 1.1) along with high positioning accuracy (±2.5 μm). It provides a wide range of adjustable parameters, including power, frequency, and scanning speed, making it ideal for systematic color marking studies. The main technical specifications of the laser system are summarized in Table 3.

**Figure 1:**
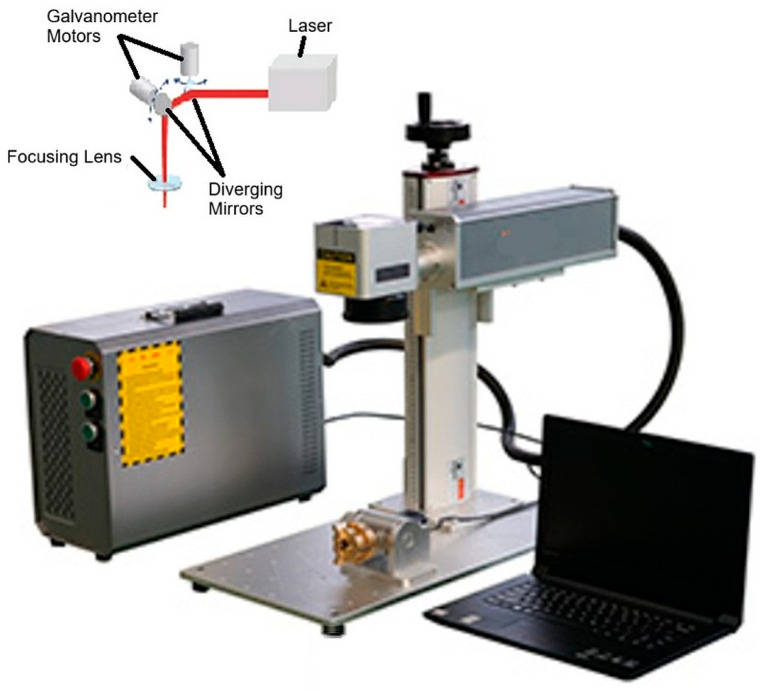
Fiber laser system used in the research.

The laser marking process is carried out by controlling the laser intensity according to the pre-selected marking data, edited in a graphical tool. Positioning and guidance of the laser radiation are achieved using a scanner. The focusing optics ensure the necessary power density in the processing area, facilitating the research process by laser scanning across the sample surface (Figure 1).

### 2.3. Experimental Methodology

**Figure 2:**
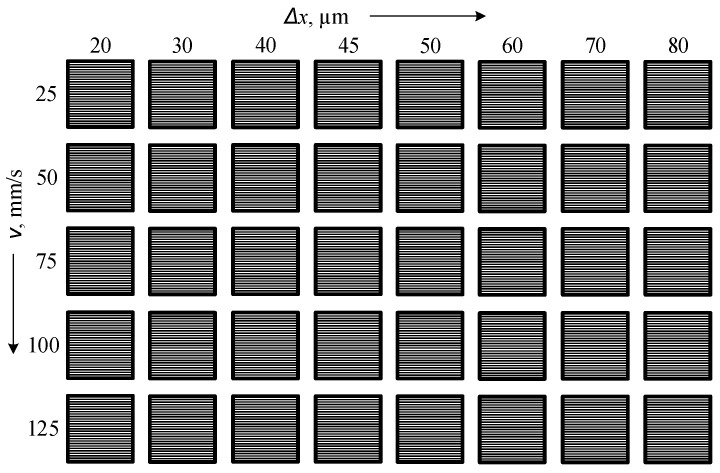
Scheme of the matrix for conducting experiments for frequences 20 kHz, 50 kHz, and 100 kHz.

To systematically study the influence of laser parameters on surface roughness and color formation, a matrix of marking experiments was designed. The matrix consisted of 5 rows and 8 squares per row (Figure 2). A raster marking method was applied, where each row was processed at different scanning speeds: *v* = 25, 50, 75, 100, and 125 mm/s, combined with raster steps Δ*x* ranging from 20 μm to 80 μm.

The influence of laser frequency was studied at three levels: 20 kHz, 50 kHz, and 100 kHz.

The effective energy density in the irradiated area for the physical process of laser interaction of radiation with matter is calculated according to Equation (1):

*E_l_* = *P*/*v*, J/m, (1)

where the average laser power *P* is constant at 20 W and *v* is the scanning speed in the range from 25 mm/s to 125 mm/s.

**Figure 3:**
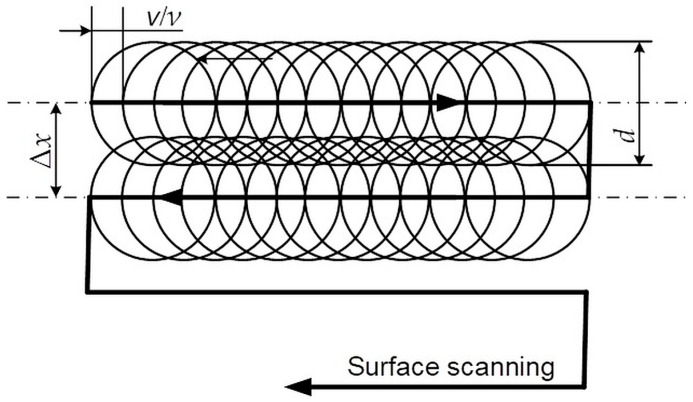
Scheme of raster laser marking of a sample with speed *v* and raster step Δ*x*.

The degree of overlap in both X and Y directions depends on the spot size, frequency, scanning speed, and raster step (Figure 3). This overlap strongly affects both the surface roughness and the resulting color tones.

The scan overlap coefficient is determined by the following formula:

*k_soc_* = (1 − Δ*x*/*d*) × 100%, (2)

where Δ*x* is the raster step in the range from 20 μm to 80 μm and the diameter of working spot, *d*, is constant at 70 μm.

### 2.4. Surface Roughness Measurements

Surface roughness of the marked zones was analyzed using a LEXT OLS5100-SAF 3D Measuring Laser Microscope (Olympus, Tokyo, Japan) for material analysis, providing high-resolution 3D imaging and precise roughness measurements. Key specifications are given in Table 4. For the purposes of the study, an optical magnification of 451× and a laser scanned area of 640 × 640 µm were used for roughness analysis.

The microscope allowed quantitative evaluation of surface topography and correlation between roughness *R_a_* and color appearance.

**Figure 4:**
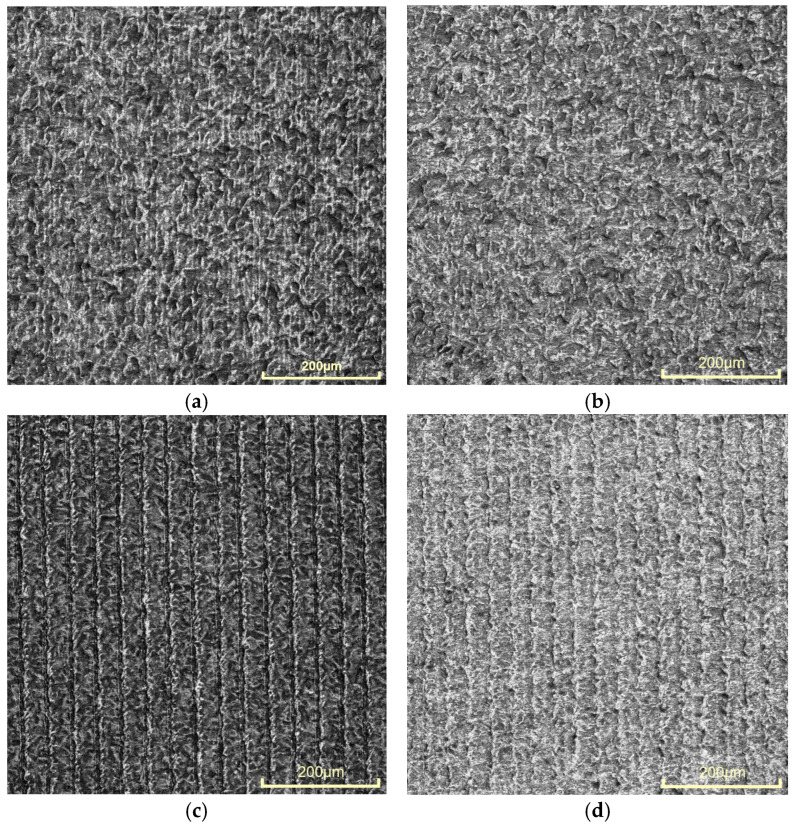
Marked fields with a fiber laser on AISI 304 stainless steel samples with the following parameters: (**a**) 20 kHz, 20 μm, and 25 mm/s; (**b**) 50 kHz, 20 μm, and 25 mm/s; (**c**) 50 kHz, 80 μm, and 25 mm/s; (**d**) 100 kHz, 70 μm, and 25 mm/s.

**Figure 5:**
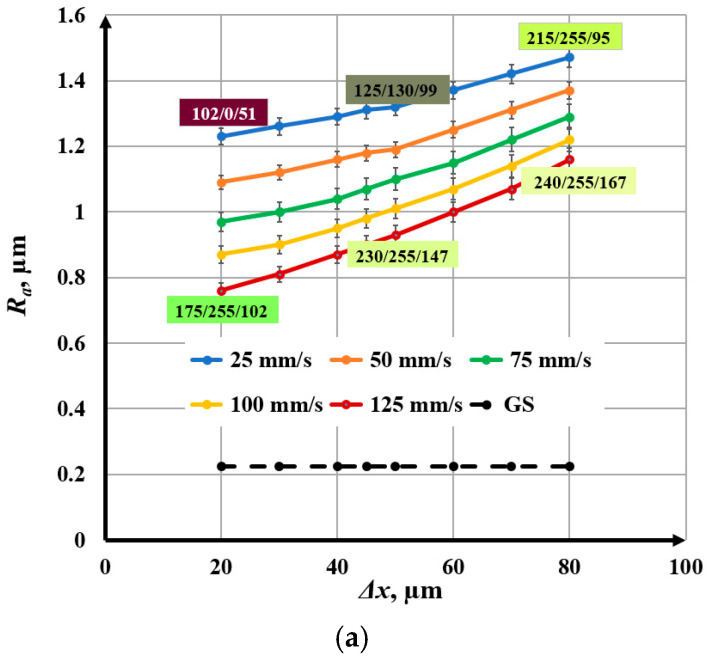

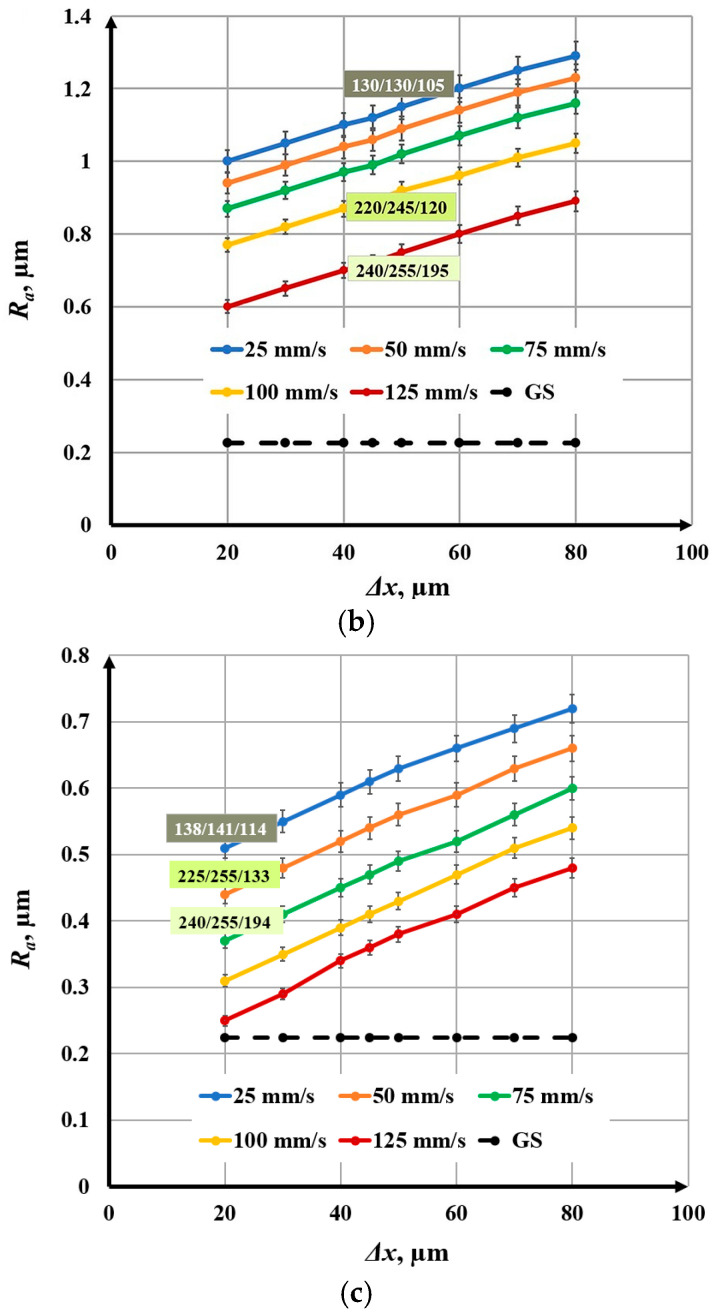
Graphics of the dependence of roughness on the raster step for five speeds: blue—25 mm/s, orange—50 mm/s, green—75 mm/s, yellow—100 mm/s, and red—125 mm/s and frequencies of (**a**) 20 kHz, (**b**) 50 kHz, and (**c**) 100 kHz. The black dotted line is the roughness of the unprocessed surface.

**Figure 6:**
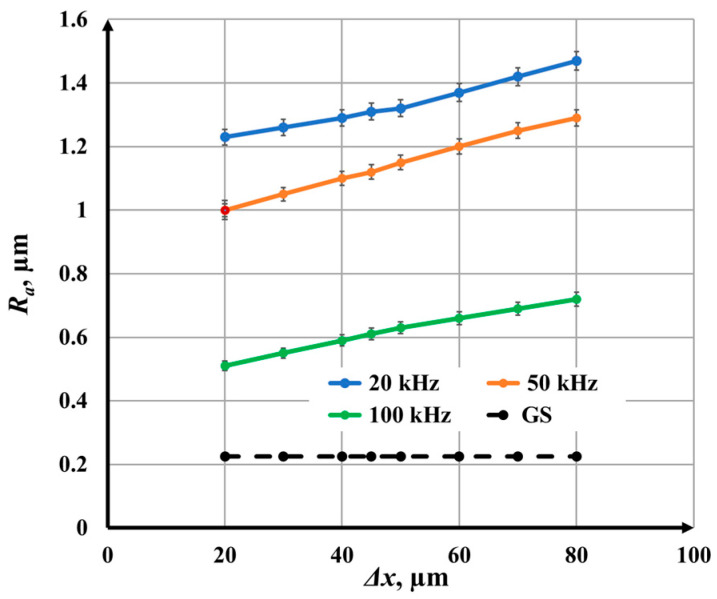
Graphics of the dependence of roughness on the raster step for speed 25 mm/s and three frequencies: blue—20 kHz; orange—50 kHz; and green—100 kHz. The black dotted line is the roughness of the unprocessed surface.

**Figure 7:**
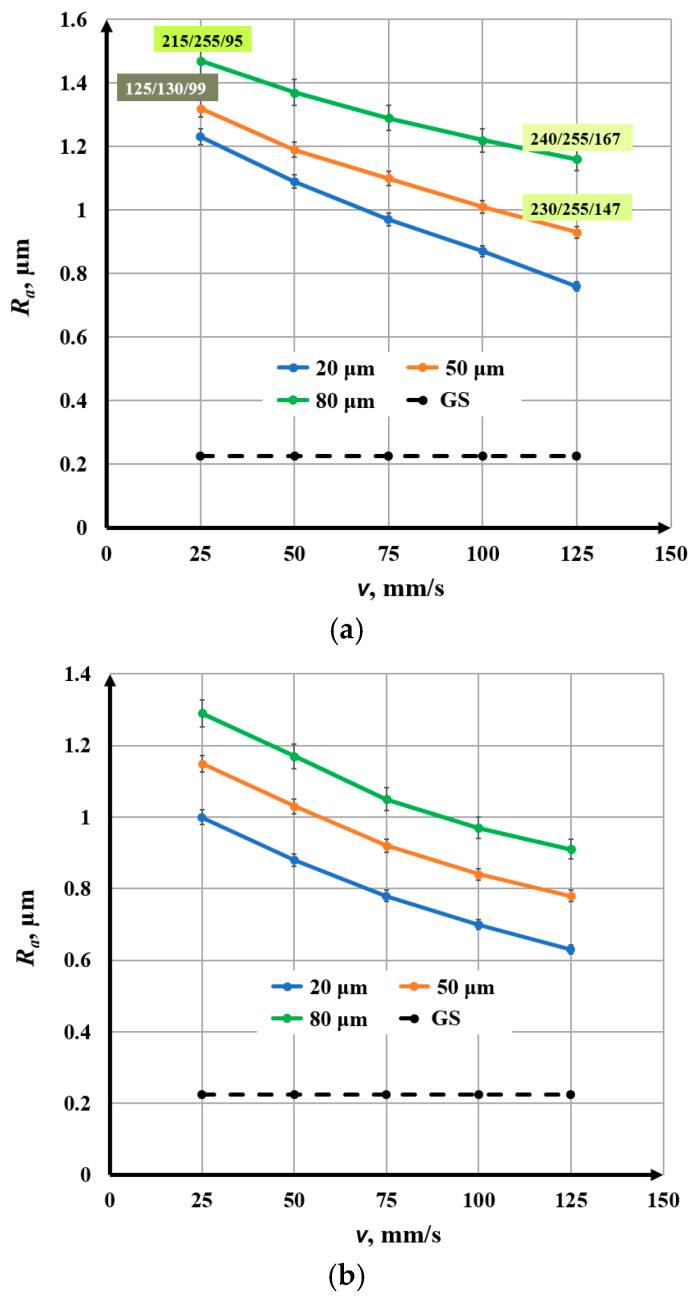

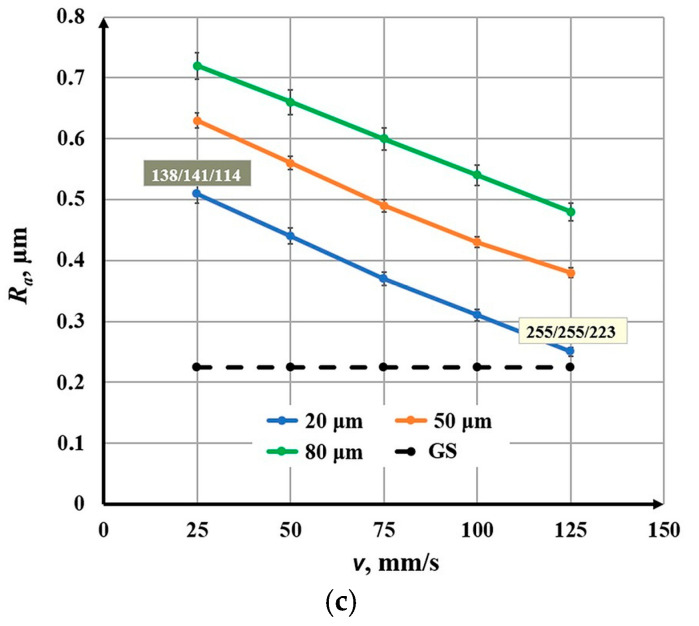
Graphics of the dependence of roughness on the speed for three raster steps: blue—20 μm; orange—50 μm; and green—80 μm and frequencies of (**a**) 20 kHz, (**b**) 50 kHz, and (**c**) 100 kHz. The black dotted line is the roughness of the unprocessed surface.

**Figure 8:**
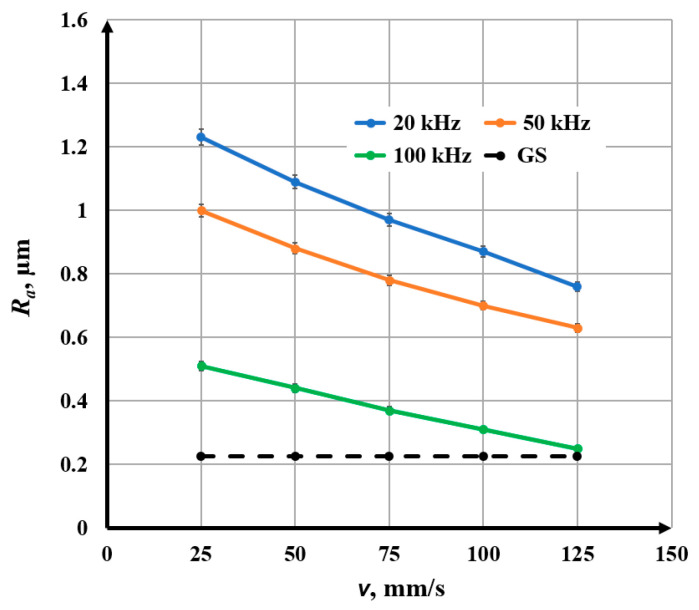
Graphics of the dependence of roughness on the speed for raster step 20 μm and three frequencies: blue—20 kHz; orange—50 kHz; and green—100 kHz. The black dotted line is the roughness of the unprocessed surface.

**Figure 9:**
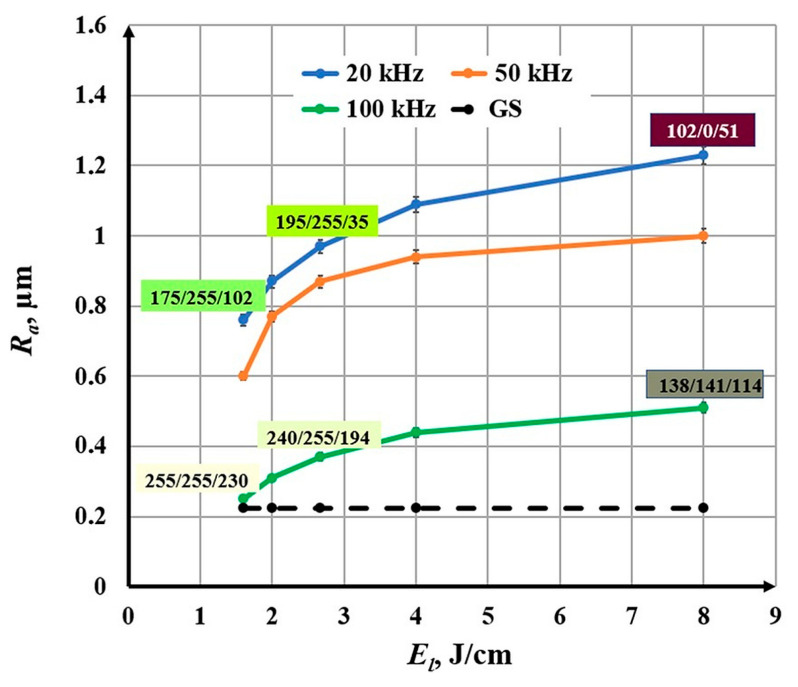
Graphics of the dependence of roughness on the linear energy density for three frequencies: blue—20 kHz; orange—50 kHz; and green—100 kHz. The black dotted line is the roughness of the unprocessed surface.

When determining the *R_a_* value for each marked area (square), 5 measurements were performed at different locations in the marked area. Then, the average value of *R_a_*, the root mean square error, and the percentage error were determined. The percentage error varied in the range from 1.1% to 2.7%. The final results are presented graphically in Figure 4, Figure 5, Figure 6, Figure 7, Figure 8 and Figure 9.

### 2.5. Measurement of Spectral Characteristics of Color-Marked Areas

Measurement of the reflectance spectra of colors obtained during laser marking of steel samples is performed using the Ocean Optics CHEM4-VIS-NIR USB4000 spectrophotometer (Ocean Optics, Inc., Dunedin, FL, USA). The spectrophotometer consists of a light source, a measuring probe, and a meter in which the incoming light is analyzed. The optical probe is a bundle of optical fibers with the same diameter (600 μm), tightly packed and enclosed in a steel tube. Using this probe, we can perform reflection measurements, covering both the specular and diffuse components of the light. The spectrophotometer measures the amount of light, converts the received electrical signal into digital form and transmits the data to the computer. It compares the received signal with the reference signal and displays the spectral characteristic and spectral coordinates.

### 2.6. Investigated Functional Dependencies in the Conducted Experiments

- The role of raster step Δ*x* on roughness R_a_ = *R_a_* (Δ*x*)
- The role of speed *v* on roughness *R_a_* = *R_a_* (*v*)
- The role of frequency *ν* on roughness (column diagram) *R_a_* = *R_a_* (*ν*)
- The role of linear energy density *E_l_* on roughness *R_a_* = *R_a_* (*E_l_*) for obtaining a certain color in the processing area
- The role of the scan overlap coefficient *k_soc_* on roughness *R_a_* = *R_a_* (*k_soc_*)

## 3. Results

### 3.1. Influence of Raster Step on Roughness

The raster step changes in the interval Δ*x* Є [20, 80] µm. Experiments refer to five values of the speed of marking: *v*_1_ = 25 mm/s, *v*_2_ = 50 mm/s, *v*_3_ = 75 mm/s, *v*_4_ = 100 mm/s, and *v*_5_ = 125 mm/s. Microscopic images of marked samples for speed *v*_1_ = 25 mm/s and different frequencies, steps, and roughness are shown in Figure 4. The parameters of the marked area in Figure 4a are frequency 20 kHz, raster step 20 μm, and roughness 1.23 μm, and the color of the marking is dark brown. The image in Figure 4b is for frequency 50 kHz, raster step 20 μm, and roughness 1.00 μm, and the resulting color is brown. The parameters of the marked area in Figure 4c are frequency 50 kHz, raster step 80 μm, and roughness 1.29 μm, and the resulting color is yellow. The marking in Figure 4d is for frequency 100 kHz, raster step 70 μm, and roughness 0.69 μm, and its color is light yellow.

The parameters that are kept constant are given in Table 5. The experimental results obtained by raster marking of a sample of AISI 304 stainless steel with a fiber laser are presented in Figure 5.

From the graphics obtained from the experimental results, the following conclusions can be drawn:

- With increasing the raster step, an increase in roughness is observed for the five studied speeds and three frequencies;
- For a frequency of 20 kHz and a speed of 25 mm/s, the roughness increases from 1.23 μm to 1.47 μm for the raster step interval from 20 μm to 80 μm. For a frequency of 20 kHz and a speed of 125 mm/s, the roughness increases from 0.76 μm to 1.16 μm for the entire studied raster step interval;
- For a frequency of 50 kHz and a speed of 25 mm/s, the roughness changes from 1.00 μm to 1.29 μm for the raster step interval from 20 μm to 80 μm. For a frequency of 50 kHz and a speed of 125 mm/s, the roughness increases from 0.60 μm to 0.89 μm for the investigated raster pitch interval;
- For a frequency of 100 kHz and a speed of 25 mm/s, the roughness varies from 0.51 μm to 0.72 μm for the raster pitch interval from 20 μm to 80 μm. For a frequency of 100 kHz and a speed of 125 mm/s, the roughness varies from 0.25 μm to 0.48 μm for the investigated raster pitch interval;
- Graphics of the dependence of roughness on the raster pitch for a speed of 25 mm/s and three frequencies: 20 kHz, 50 kHz, and 100 kHz are presented in Figure 6. For a frequency of 20 kHz, the roughness is about 15% greater than that for 50 kHz and 3–4 times greater than that for a frequency of 100 kHz.

### 3.2. Influence of Speed on Roughness

The speed varies in the interval *v* Є [25, 125] mm/s. The experiments were conducted for three raster steps: Δ*x*_1_ = 20 μm, Δ*x*_2_ = 50 μm, and Δ*x*_3_ = 80 μm. The parameters that do not change during the experiments are given in Table 5. From the obtained graphics in Figure 7, the following conclusions can be drawn:

- With increasing speed, a nonlinear decrease in roughness is observed for the three studied raster steps and the three frequencies;
- For a frequency of 20 kHz and a raster step of 20 μm, the roughness decreases from 1.23 μm to 0.76 μm for the speed interval from 25 mm/s to 125 mm/s. For a frequency of 20 kHz and a raster step of 80 μm, the roughness decreases from 1.47 μm to 1.16 μm for the entire studied speed interval;
- For a frequency of 50 kHz and a raster step of 20 μm, the roughness changes from 1.00 μm to 0.60 μm for the studied speed interval. For a frequency of 50 kHz and a raster step of 50 μm, the roughness decreases from 1.29 μm to 0.89 μm for the studied speed interval;
- For a frequency of 100 kHz and a raster step of 20 μm, the roughness varies from 0.51 μm to 0.25 μm for the studied speed interval. For a frequency of 100 kHz and a raster step of 80 μm, the roughness varies from 0.72 μm to 0.48 μm for the studied speed interval;
- The graphics of the dependence of roughness on speed for a raster step of 20 μm and three frequencies: 20 kHz, 50 kHz, and 100 kHz are presented in Figure 8. For the frequency of 20 kHz, the roughness is about 20% greater than that for 50 kHz and about 2.5 times greater than that for the frequency of 100 kHz.

### 3.3. Influence of the Linear Energy Density on the Roughness

The linear energy density varies in the interval *E_l_* Є [1.60, 8.00] J/cm. The experiments were conducted for three frequencies: *ν*_1_ = 20 kHz, *ν*_2_ = 50 kHz, and *ν*_3_ = 100 kHz. The following conclusions can be made from the obtained graphics in Figure 9:

- With increasing linear energy density, a nonlinear increase in roughness is observed for the three studied frequencies;
- For a frequency of 20 kHz, the roughness increases from 0.76 μm to 1.23 μm for the linear energy density interval from 1.60 J/cm to 8.00 J/cm;
- For a frequency of 50 kHz, the roughness varies from 0.60 μm to 1.00 μm for the studied linear energy density interval;
- For a frequency of 100 kHz, the roughness increases from 0.25 μm to 0.51 μm for the studied linear energy density interval.

### 3.4. Influence of the Scan Overlap Coefficient on the Roughness

**Figure 10:**
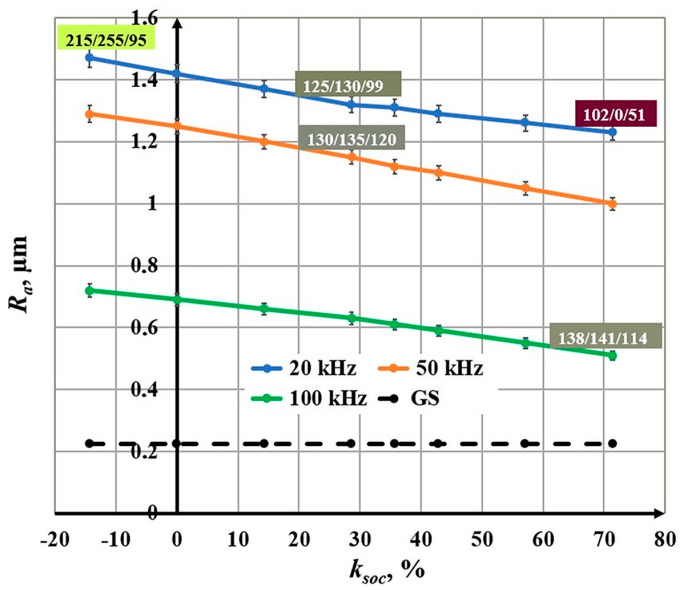
Graphics of the dependence of roughness on the scan overlap coefficient for three frequencies: blue—20 kHz; orange—50 kHz; and green—100 kHz. The black dotted line is the roughness of the unprocessed surface.

The scanning overlap coefficient changes in the interval *k_soc_* Є [−14.3, 71.4] %. The experiments were conducted for three frequencies: *ν*_1_ = 20 kHz, *ν*_2_ = 50 kHz, and *ν*_3_ = 100 kHz. The following conclusions can be drawn from the obtained graphics in Figure 10:

- With an increasing scanning overlap coefficient, a linear decrease in roughness is observed for the three studied frequencies;
- For a frequency of 20 kHz, the roughness decreases from 1.47 μm to 1.23 μm for the scanning overlap coefficient interval from 10.4% to 71.4%;
- For a frequency of 50 kHz, the roughness changes from 1.29 μm to 1.00 μm for the studied scanning overlap coefficient interval;
- For a frequency of 100 kHz, the roughness decreases from 0.72 μm to 0.51 μm for the studied scanning overlap coefficient interval.

### 3.5. Influence of Frequency on Roughness

**Figure 11:**
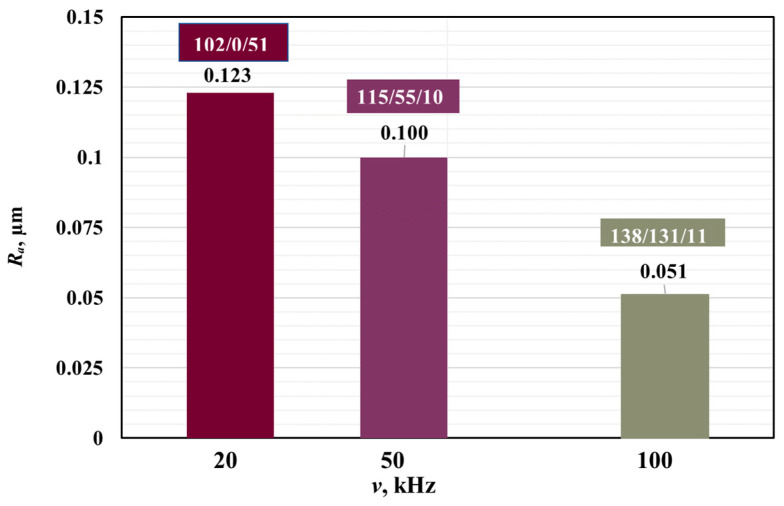
Diagram of the dependence of roughness on the frequency.

A tabular graphic is presented in Figure 11 for the three studied frequencies 20 kHz, 50 kHz, and 100 kHz, with all three zones marked at a speed of 25 mm/s and a raster step of 20 μm. With increasing frequency, a decrease in the roughness of the marking is observed, and its color changes from dark color (brown) to light color (yellow).

### 3.6. Relationship Between Technological Parameters and Color Contrast

From the results obtained, some of which are presented in Table 6, we identified some relationships and trends between technological parameters and roughness and color contrast:

- With increasing raster step, the roughness of the marked areas increases and a change in the marking from dark (brown color) to light (yellow color) is observed when the other technological parameters are kept constant;
- With increasing speed, the roughness of the marked areas decreases and a change in the marking from dark to light is observed;
- With increasing frequency, the roughness of the marked areas decreases and a lightening of the marking is observed;
- Increasing the linear energy density leads to an increase in the roughness of the marked areas and a darkening of the marking;
- Increasing the scanning overlap coefficient leads to an increase in the roughness of the marked areas and a darkening of the marking.

## 4. Conclusions

The conducted experiments demonstrate that laser processing parameters exert a direct and quantifiable influence on both the surface roughness and color contrast of AISI 304 stainless steel. Increasing the raster pitch from 20 µm to 80 µm causes a steady rise in roughness (e.g., from 1.23 µm to 1.47 µm at 20 kHz and 25 mm/s) and a corresponding shift from dark brown to lighter yellow tones. In contrast, higher scanning speeds (25–125 mm/s) result in a nonlinear decrease in roughness (from 1.23 µm to 0.76 µm at 20 kHz and Δ*x* = 20 µm), accompanied by progressive surface lightening. The laser frequency proved critical: increasing it from 20 kHz to 100 kHz reduced roughness approximately threefold (from 1.23 µm to 0.25 µm at a 20 µm raster step and 125 mm/s) and produced bright yellow hues. Likewise, greater linear energy density (1.60–8.00 J/cm) led to a nonlinear increase in roughness and darker coloration, whereas a higher overlap coefficient reduced roughness and maintained lighter colors.

These results confirm that stable and reproducible interference colors are closely related to the controlled nanostructuring of the surface. Thin oxide layers are formed by uniformly heating the surface with a laser beam in the presence of oxygen in the laser marking zone. The temperature increase resulting from the laser action enhances the diffusion flux of oxygen and the rate of the oxidation reactions taking place. The process is influenced by the focal spot diameter, power, marking speed, raster pitch, frequency, pulse duration, etc., some of which are investigated in the present work. The established correlations clarify the mechanisms of color formation and roughness modulation and define technological windows for achieving targeted chromatic effects. This knowledge supports the industrial application of color laser marking, combining aesthetic differentiation with functional advantages such as improved corrosion resistance, hydrophobicity, and antibacterial properties.

Future work will focus on evaluating the functional performance of colored surfaces obtained under optimized parameters—specifically corrosion resistance, hydrophobicity, and durability—to further assess their practical potential. These investigations will strengthen the structural and aesthetic insights established here and advance the development of functional surface engineering. In the next stage, the research will be extended to color marking of other stainless steels and chromium-based materials to broaden the applicability of the findings.

## Figures and Tables

**Table 1:** Chemical composition of AISI 304 stainless steel [19].

Chemical Element	Content, %	Chemical Element	Content, %
C	<0.07	Si	<1.0
Cr	17.5–19.5	P	<0.045
Ni	8.0–10.5	S	<0.03
Mn	<2.0	Fe	Balance
N	<0.10		

**Table 2:** Basic physical properties of AISI 304 stainless steel [20].

Parameter	Value
Coefficients of thermal conductivity *k*, W/(m.K)	16.2
Density *ρ*, kg/m^3^	7850
Specific heat capacity *c*, J/(kg.K)	504
Coefficients of thermal diffusivity *a*, m^2^/s	4.09 × 10^−6^

**Table 3:** Parameters of laser system used in the research.

Parameter	Value	Parameter	Value
Wavelength *λ*, nm	1064	Pulse energy *E_p_*, mJ	0.08–1.00
Average power *P*, W	20	Pulse power *P_p_*, kW	0.80–10.0
Frequency *ν*, kHz	20–250	Beam quality *M*^2^	<1.1
Pulse duration *τ*, ns	100	Scan speed *v*, mm/s	1–20,000
Positioning accuracy	±2.5 μm	Efficiency, %	40
Diameter in focus *d*, µm	70		

**Table 4:** Specifications of the OLS5100 laser microscope.

Parameter	Value	Parameter	Value
Total magnification	54×–17,280×	Measurement accuracy	±1.5%
Field of view	16 μm–5120 μm	Laser wavelength	405 nm
Display resolution	1 nm	Laser source power	0.95 mW
Max measuring points	4096 × 4096 pixels	Optical zoom	54×–2160×

**Table 5:** Parameters that do not change during experiments.

Parameter	Value	Parameter	Value
Power *P*, W	20	Number of repetition *P_p_*, kW	1
Frequency *v*, kHz	20, 50, 100	Defocusing Δ*f*	0
Pulse duration *τ*, ns	100	Diameter of working spot *d*, µm	70

**Table 6:** Technological parameters and R/G/B coordinates of the resulting color marking.

*ν*, kHz	*v*, mm/s	Δ*x*, μm	*k_soc_*, %	*E_l_*, J/cm	*R_a_*, μm	*R/G/B*
20	25	20	71.4	8.00	1.23	102/0/51
20	25	50	28.6	8.00	1.32	125/130/99
20	25	80	−14.3	8.00	1.47	215/255/95
20	75	20	71.4	2.67	0.97	195/255/35
20	75	50	28.6	2.67	1.10	215/255/130
20	125	20	71.4	1.60	0.76	175/255/102
20	125	50	28.6	1.60	0.93	230/255/147
20	125	80	−14.3	1.60	1.16	240/255/167
50	25	50	28.6	8.00	1.15	130/135/120
50	50	50	28.6	4.00	1.09	210/240/107
50	100	50	28.6	2.00	0.92	220/245/120
50	125	50	28.6	1.60	0.75	240/255/195
100	25	20	71.4	8.00	0.51	138/141/114
100	50	20	71.4	4.00	0.44	225/255/133
100	75	20	71.4	2.67	0.37	240/255/194
100	125	20	71.4	1.60	0.25	255/255/223

## Data Availability

The original contributions presented in this study are included in the article. Further inquiries can be directed to the corresponding author.
